# Acoustic rhinometry and video endoscopic scoring to evaluate postoperative outcomes in endonasal spreader graft surgery with septoplasty and turbinoplasty for nasal valve collapse

**DOI:** 10.1186/s40463-016-0115-9

**Published:** 2016-01-12

**Authors:** Bree Erickson, Robert Hurowitz, Caroline Jeffery, Khalid Ansari, Hamdy El Hakim, Erin D. Wright, Hadi Seikaly, Sam R. Greig, David W. J. Côté

**Affiliations:** Department of Surgery, Division of Otolaryngology – Head and Neck Surgery, University of Alberta, 1E4 Walter C Mackenzie Centre, 8440-112 Street NW, Edmonton, AB T6G 2B7 Canada; Faculty of Medicine and Dentistry, University of Alberta, Edmonton, AB Canada

**Keywords:** Internal nasal valve collapse, Spreader graft, Septoplasty, Turbinoplasty, Acoustic rhinometry

## Abstract

**Background:**

Nasal obstruction is a common complaint seen by otolaryngologists. The internal nasal valve (INV) is typically the narrowest portion of the nasal cavity, and if this area collapses on inspiration the patient experiences significant symptoms of nasal obstruction. The nasal obstruction is further compounded if the INV is narrower than normal. Previous studies have evaluated the effectiveness of techniques to alleviate structural nasal obstruction, but none have looked specifically at spreader grafts measured by acoustic rhinometry or validated grading assessment of dynamic INV collapse. Our objective was to evaluate the application of acoustic rhinometry coupled with visual endoscopic grading of the INV, and validated subjective measurements, in patients undergoing endonasal spreader graft surgery with septoplasty and turbinoplasty.

**Methods:**

This is a prospective clinical study conducted within a tertiary care rhinoplasty practice. Patients undergoing septoplasty and bilateral inferior turbinoplasty with bilateral endonasal spreader graft placement for observed internal nasal valve collapse were recruited. Baseline, early and intermediate postoperative measures were obtained. The primary outcome was grading of the INV collapse on video endoscopy. Secondary outcomes included cross-sectional area at the INV measured by acoustic rhinometry, subjective Nasal Obstruction Symptom Evaluation (NOSE) and Sino-Nasal Outcome Tool (SNOT-22) scores.

**Results:**

A total of 17 patients, average age of 34.5 ± 12.2 years, undergoing septoplasty, bilateral endonasal spreader grafts, and bilateral turbinoplasty were included in the study. Postoperative measurements were performed at an average of 8.1 ± 1.6 weeks and 17.7 ± 4.2 weeks. Patients had significant improvement for INV collapse grading, cross-sectional area, NOSE and SNOT-22 scores in both the early and intermediate follow up. Endoscopic grading had moderate inter-rater agreement (κ = 0.579) and average intra-rater agreement (κ = 0.545).

**Conclusions:**

This study is the first to demonstrate a statistically significant improvement of objective measurement of internal nasal valve function, both static and dynamic, and subjective improvements. This supports endonasal cartilagenous spreader grafts with septoplasty and inferior turbinoplasty for patients with nasal obstruction with internal nasal valve collapse.

## Background

Nasal obstruction is a common complaint of patients seeking consultation with otolaryngologists [1]. The internal nasal valve (INV) is typically the narrowest portion of the nasal cavity and is bounded by the septum, the upper lateral cartilage (ULC), the inferior turbinate, and the nasal floor. A normal INV angle is between 10 and 15°, and a smaller angle can result in a predisposition to symptoms of nasal obstruction [2]. Collapse of the INV on inspiration can cause significant nasal obstruction. In fact, based on the Bernoulli principle, the degree of narrowing of the INV directly correlates to the tendency to collapse and obstruct.

The static area of the INV can be enlarged through various techniques depending on the cause of narrowing. Septoplasty removes and straightens obstruction caused by the nasal septum. Inferior turbinoplasty reduces the bulk of the inferior turbinates, especially at the turbinate head, where it is a contributing boundary of the INV. Endonasal spreader graft placement is a procedure used to address INV collapse [3]. The technique involves harvesting cartilage and creating grafts approximately 2–3 mm in width and 2 mm in height and 7–10 mm in length. The graft is placed into a submucosal pocket inferior to the superior edge of the ULC, running dorsally up the length of the ULC. The intent is to stent open the INV angle in order to prevent collapse during inspiration [3].

Studies evaluating the effectiveness of nasal surgery techniques to alleviate structural nasal obstruction often lack objective evidence. In particular, no study has looked specifically at endonasal spreader graft placement [4–9]. Our objective was to evaluate the utility of acoustic rhinometry and visual endoscopic grading of the INV, as well as subjective measurements, in patients undergoing spreader graft surgery with septoplasty and turbinoplasty.

## Methods

Prior to commencement of this study, ethics approval was obtained from the University of Alberta Health Research Ethics Board (Pro00041956). Informed consent to participate in this study was obtained from each of the participants. Inclusion criteria included patients over the age of 17 seen in a single tertiary care otolaryngology practice. Patients’ primary complaint was nasal obstruction and all were diagnosed with structural causes of nasal obstruction, including septal deviation and internal nasal valve collapse. Patients were excluded if they required revision septoplasty or formal septorhinoplasty. Other exclusion criteria included concomitant diagnosis of other causes of nasal obstruction requiring additional adjunct procedures (e.g. external nasal valve collapse, nasal polyposis, etc.). Surgical method involved a standard submucosal resection of the septum with placement of endonasal spreader grafts as previously described [3]. Intraturbinal turbinoplasty was performed in all patients using a Medtronic microdebrider with turbinoplasty blade as described by Lee in 2013 [10].

The primary outcome was grading of the INV collapse on video endoscopy. Secondary outcomes included cross-sectional area at the INV measured by acoustic rhinometry protocol, subjective Sino-Nasal Outcome Tool (SNOT-22) and Nasal Obstruction Symptom Evaluation (NOSE) scores. Patients underwent preoperative measurements as well as early and intermediate postoperative measurements at 6 to 12 weeks and at 12 to 16 weeks, respectively.

Acoustic rhinometry was performed with the A1 Acoustic Rhinometer (GM Instruments Ltd., Kilwinning, Scotland) utilizing recommendations as set out by the consensus report on acoustic rhinometry developed by Clement et al. in 2005 [11]. Acoustic rhinometry is a technique used to measure the cross-sectional area of the nasal cavity as a function of the distance into the nasal cavity from the nasal sill. This measurement is performed by analyzing the amplitude of the reflection of sound waves projected into the nose. The minimum cross-sectional area identified within the first 3 cm of the nose typically corresponds with the INV. A single experienced technician performed the measurements. Topical spray consisting of 0.1 % xylometazoline and 4 % lidocaine was administered 5 min prior to measurement. Nosepiece sizing was chosen to provide an appropriate acoustic seal for each patient without altering nasal anatomy. Measurements were performed during a breathing pause by the patient. Three repeated measurements were performed on each side and the minimum cross-sectional area value was averaged from the readings.

Rigid endoscopy was next performed, using 0° 4 mm Olympus rigid endoscope and video recorded. The patient was asked to inhale repetitively as the video was recorded examining bilateral INV.

Finally, patients were asked to complete the NOSE scale and SNOT-22 at the time of their measurements pre- and postoperatively. The Nasal Obstruction Symptom Evaluation (NOSE) Scale was developed and validated by Stewart et al. in 2004 [12]. This survey was initially developed for assessment of septoplasties but can be used for any corrective technique for nasal obstruction of varying cause. The Sino-Nasal Outcome Tool (SNOT-22), however, was developed for patients with chronic sinusitis and therefore not limited to nasal obstruction alone, although it has been shown to be an effective measurement in patients undergoing septoplasty [13, 14].

Collapse of the INV occurs when the lateral nasal wall moves inwards during inspiration. This dynamic change in INV dimensions has been described as a percentage of full collapse with a grading scheme developed by Most et al. in 2008 and validated in 2013 by Tsao et al. [15, 16]. The grading scheme consists of grades 1 through 3, as <33 %, 33–66 % collapse, and >66 % collapse towards the septum respectively. Four independent otolaryngologists performed analysis of the video endoscopy. The videos were divided into preoperative, postoperative, right and left, and then randomized. The reviewers were blinded to the patients and operative status and asked to grade the degree of INV collapse based on Tsao et al. validated grading method published in 2013 [16]. The final grading of each video was calculated by averaging the grades from the four reviewers.

### Statistical methods

Statistical analysis of measurements was performed using SPSS version 22.0 (SPSS Inc., Chicago IL, USA). Descriptive statistics were performed to assess the data. Test for normality on difference of data sets was calculated using Kolmogorov-Smirnov and Shapiro-Wilk tests. Paired t-test was performed to compare the preoperative and postoperative data for video grading scores, acoustic measurements of INV cross-sectional area, and patient-completed surveys NOSE and SNOT-22 scores. Statistical significance was defined as *p* <0.05. Fleiss kappa calculation for inter- and intra-rater agreement was performed using the online calculator developed by Geertzen in 2012 for inter-rater agreement with multiple raters [17].

## Results

A total of 17 patients undergoing septoplasty, bilateral endonasal spreader grafts, and bilateral turbinoplasty were included in the study. Demographics included a male to female ratio of 16:1 and an average age of 34.5 ± 12.2 years. Average early follow up time was 8.1 ± 1.6 weeks and 17.7 ± 4.2 weeks for intermediate follow up. No postoperative complications occurred in any of the patients. Measurements for early and intermediate follow up time were obtained for 14 and 12 patients respectively. Preoperative and postoperative measurement means are presented in Table 1. All sets of data, were normally distributed, and therefore preoperative and postoperative comparisons were performed with paired t-test. Calculations comparing preoperative and postoperative data sets for all variables were shown to have a significant improvement (Tables 2 and 3). Inter-rater agreement was found to be moderate between the four graders, with a kappa value of 0.579. Intra-rater agreement was also moderate with an average kappa value of 0.545 (κ_1_ = 0.610, κ_2_ = 0.534, κ_3_ = 0.574, κ_4_ = 0.461).

**Table 1:** Preoperative and postoperative mean values for measured variables

Variable	Preoperative (*N* = 17)			Early postoperative (*N* = 14)			Intermediate postoperative (*N* = 12)		
	Mean	Range	SD	Mean	Range	SD	Mean	Range	SD
INV collapse grading	2.23	1–3	0.67	1.37	1–2.5	0.46	1.54	1–3	0.70
INV cross-sectional area (cm^2^)	0.519	0.02–1.10	0.278	0.614	0.19–1.32	0.277	0.552	0.17–1.01	0.230
NOSE	14.0	10–20	3.3	5.9	2–10	2.9	7.7	0–15	4.5
SNOT-22	34.1	13–71	16.5	16.9	1–55	15.4	22.7	1–57	19.8

*SD* standard deviation, *INV* internal nasal valve, *NOSE* nasal obstruction symptom evaluation scale, *SNOT* sino-nasal outcome tool

**Table 2:** Comparison of preoperative and early postoperative values (paired t-test)

Variable	Mean of difference	95 % CI	Significance
INV collapse grading	0.857	0.542–1.172	<0.001
INV cross-sectional area (cm^2^)	0.095	0.001–0.189	0.047
NOSE	8.1	6.1–10.2	<0.001
SNOT-22	17.3	10.2–24.4	<0.001

*CI* confidence interval, *INV* internal nasal valve, *NOSE* nasal obstruction symptom evaluation scale, *SNOT* sino-nasal outcome tool

**Table 3:** Comparison of preoperative and intermediate postoperative values (paired t-test)

Variable	Mean of difference	95 % CI	Significance
INV collapse grading	0.859	0.528–1.190	<0.001
INV cross-sectional area (cm^2^)	0.075	0.005–0.145	0.036
NOSE	6.9	4.9–8.9	<0.001
SNOT-22	19.1	12.5–25.7	<0.001

*CI* confidence interval, *INV* internal nasal valve, *NOSE* nasal obstruction symptom evaluation scale, *SNOT* sino-nasal outcome tool

## Discussion

This is the first study that provides objective evidence of the utility of endonasal spreader grafts, when performed in conjunction with nasal septoplasty and inferior turbinoplasty, in addressing the internal nasal valve. Both static and dynamic measurements were obtained along with subjective symptomatic improvement. Several studies have previously investigated objective measures of improvement with spreader graft surgery using cadaver models [4, 5]. Huang et al., in 2006, studied an endoscopic approach to spreader grafts on cadaveric heads using 8 specimens. They found significantly improved nasal valve area (mean change 0.28 cm^2^) using acoustic rhinometry measurements. Craig et al., in 2014, also performed spreader graft placement on 6 cadaveric heads and also found a significant improvement in INV area (mean change 0.10 cm^2^). Rigid nasal endoscopy was used in this study, but only to classify the INV as normal or narrow, not as a means to measure improvement. Of note, this study did find that there was greater improvement in the INV with narrow classification (cross-sectional area less than 0.50 cm^2^) than in those with normal based on a normal classification. A statistically significant 51 % improvement was found for narrow INV, whereas only a 1 % improvement for normal preoperative INV was found. Neither of these above mentioned studies were able to assess dynamic changes or subjective assessments as these were performed in cadaveric models. Of note, there has been no establishment of a clinically significant improvement in INV area in the current literature.

Few studies have examined both the objective and subjective outcomes of surgery for nasal obstruction. Haavisto et al., in their 2012 paper, examined the use of acoustic rhinometry and rhinomanometry as well as a visual analogue scale to evaluate improvement in unilateral nasal obstruction in 30 patients undergoing septoplasty [6]. Objective measure of patients improved significantly and a trend toward improvement in patient satisfaction was also found, though not statistically significant. Mengi et al., in 2011, however did show a significant improvement in NOSE scores, minimal cross-sectional area measured by acoustic rhinometry, and nasal resistance values measured by rhinomanometry after septoplasty in 44 patients [7].

Edizer et al. evaluated 26 patients undergoing septorhinoplasty for objective improvement in nasal airway using acoustic rhinometry and subjective improvements using a 10-point visual analog scale [8]. Although the patients all underwent septorhinoplasty, not all had preoperative complaints of nasal obstruction. The study found significant improvement in symptom scoring but not in cross-sectional area. Zoumalan et al., in 2012, also evaluated objective and subjective measurements of 31 septorhinoplasty patients [9]. This study also used acoustic rhinometry and a 10-point rating scale for the measurements. Thirteen patients underwent spreader grafts, but it is unclear as to what other techniques were used in their septorhinoplasty procedures. Our study is the first to provide data on a procedure specifically aimed at correcting the INV static area and dynamic collapse.

Our study highlights not only the utility of endonasal spreader grafts with septoplasty and turbinoplasty, but also the significant quality of life issues that accompany nasal obstruction due to INV collapse. In 2009, Gillett et al. showed that normal subjects who are free of sinonasal disease have an average SNOT-22 score of 7 [18]. The patients in the study had preoperative average NOSE and SNOT-22 scores of 14.1 and 34.8 respectively, both of which are significant for symptomatic disease.

A potential limitation of this study is the reliability of acoustic rhinometry. Various studies have shown varying degrees of reliability of acoustic rhinometry as compared to computed tomography scans and magnetic resonance imaging, assessing both cross-sectional area and volume of the nasal cavity [19–22]. These studies have shown significant correlation between acoustic rhinometry and imaging for the anterior portion of the nose but not the posterior. This finding was confirmed in a more recent study using high-resolution computed tomography scanning by Numminen et al. [23]. A statistically significant correlation was found between the minimum cross-sectional areas in the first 10 mm and 11–40 mm of the nasal cavity. There was a weaker correlation in the posterior portion of the nose. A well-defined measurement protocol, similar to the one used in our study, was employed for acoustic measurement. Using a single experienced technician, appropriately sized nasal coupling pieces, and consistent technique strengthened our measurement reliability. In addition, the study specifically examines the most anterior portion of the nasal cavity, which is the most accurately measured area.

The inter-rater agreement for this study was calculated as fair to good, with a kappa value of 0.579. This is less robust than the kappa of 0.77 found by the validating paper for this grading scheme. It will be interesting in the future to observe what other groups are able to attain for inter-rater agreement for this grading scheme to better determine its clinical utility.

Finally, due to the combined techniques of septoplasty and turbinoplasty with the endonasal spreader graft placement, it is not possible to determine how much of the objective and subjective success is attributable to the spreader grafts alone. Potentially, the increase in cross-sectional area may be more attributable to the correction of septal deviation and reduction of the turbinates whereas the decrease in INV collapse seen on video endoscopy should be attributable to the spreader graft placement. However, the increased INV area may improve the degree of INV collapse due decreased Bernoulli effect.

This study included a specific group of patients who demonstrated nasal obstruction from both static and dynamic causes. Although previously Mengi et al. found significant increase in INV area from septoplasty alone, this is not applicable to our patient population that had demonstrated INV collapse [7]. As described by Rhee et al. in their 2010 consensus statement on diagnosis and management of nasal valve compromise there are thought to be some cases where septoplasty and/or turbinate surgery can be used to treat nasal valve collapse without surgery to support the lateral wall [24]. Ideally a randomized controlled trial of septoplasty and turbinoplasty with and without spreader graft placement could better show the effectiveness of spreader graft placement. This future research would be justified to determine which cases of INV collapse truly require endonasal spreader grafts for surgical correction.

Observation of the demographics of the patients in this study reveals a significant preponderance of male patients. This preponderance is seen in the study by Haavisto et al. addressing surgical correction of nasal obstruction but has not been noted elsewhere [6]. Further review of patient populations may reveal that male patients either exhibit more nasal obstruction or seek surgical correction of this obstruction more often than their female counterparts.

## Conclusion

This study provides an agreement of objective measurement of internal nasal valve function, both static and dynamic, with subjective patient improvement supporting endonasal cartilagenous spreader grafts in combination with septoplasty and inferior turbinoplasty as a safe and effective approach for patients complaining of nasal obstruction with internal nasal valve collapse.

### Ethics approval

Prior to commencement of this study ethics approval was obtained from the University of Alberta Health Research Ethics Board.
